# Chick Early Amniotic Fluid (ceAF) Deters Tumorigenesis via Cell Cycle Arrest and Apoptosis

**DOI:** 10.3390/biology11111577

**Published:** 2022-10-27

**Authors:** Mashaal Ahmad, Jia Yu, Sha Cheng, Zara Ahmad Khan, Yan Luo, Heng Luo

**Affiliations:** 1 State Key Laboratory of Functions and Applications of Medicinal Plants, Guizhou Medical University, Guiyang 550014, China; 2 Department of Biochemistry and Cancer Institute of the Second Affiliated Hospital (Key Laboratory of Cancer Prevention and Intervention of China National MOE), Zhejiang University School of Medicine, Hangzhou 310058, China; 3 The Key Laboratory of Chemistry for Natural Products of Guizhou Province and Chinese Academy of Sciences, Guiyang 550014, China; 4 Department of Anatomy, School of Basic Medical Sciences, Guizhou Medical University, Guiyang 550014, China; 5 State Key Laboratory of Oncogenes and Related Genes, Institute for Personalized Medicine, School of Biomedical Engineering, Shanghai Jiao Tong University, Shanghai 200030, China

**Keywords:** chick early amniotic fluid, tumorigenesis, breast cancer, colon cancer apoptosis, cell cycle arrest

## Abstract

**Simple Summary:**

Amniotic fluids have been utilized extensively in several clinical medicine applications, including cutaneous wound healing, myocardial infarctions, and regeneration of organs, as they are the source of many key components, signaling molecules, cytokines, and peptides. Here in this study, we reported the applications of chick early amniotic fluid (ceAF) against tumorigenesis and showed how ceAF contributes to significant inhibition of metastasis of cancer cells, including breast cancer and colon carcinoma cell lines, and compared with a regular cell line. Our study also highlights the efficacy of ceAF in a dose-dependent manner in a different type of cancers. The in vivo study and biochemical parameters of the tumor xenografts also provided similar findings indicating the promising therapeutic potentials of ceAF.

**Abstract:**

In recent years, amniotic fluids have gained attention in cancer research. They have an influential role in protecting embryos against several anomalies. Chick early amniotic fluid (ceAF)—amniotic fluid isolated from growing chicken—has been used in many other studies, including myocardial infarctions and skin regeneration. In this study, we employed ceAF’s promising therapeutic applications against tumorigenesis in both in vitro and in vivo studies. We selected three robust proliferating tumor cell lines: BCaP37, MCF7, and RKO. We found that selective dosage is required to obtain maximum impact to deter tumorigenesis. ceAF not only disrupted the uniform colonies of tumor cell lines via disturbing mitochondrial transmembrane potential, but also arrested many cells at growing G1 state via working agonistically with aphidicolin. The significant inhibition of tumor metastasis by ceAF was indicated by in vivo models. This leads to apoptosis analysis as verified by annexin-V staining stays and immunoblotting of critical proteins as cell cycle meditators and apoptosis regulators. Not only on the protein level, but we also tested ceAF’s therapeutic potentials on mRNA levels as indicated by quantitative real-time PCR summarizing the promising role of ceAF in deterring tumor progression. In conclusion, our study reveals the potent role of ceAF against tumorigenesis in breast cancer and colon carcinoma. Further studies will be required to determine the critical components present in ceAF and its purification to narrow down this study.

## 1. Introduction

Despite substantial progress in treating different types of cancer in the past decades, it remains a major universal health problem [1]. The number of deaths can be reduced by early detection and preventive measures. Moreover, the expansion of new targeted-based therapies offers specific opportunities to deal with the current increased ratio of different types of cancer. However, the significant problem for developing novel treatment regimens is the critical issue of transitioning scientific knowledge from bench to bedside, probably due to poor recapitulation of the patient’s tumor study [2]. As a result, many drugs that perform well in the cancer case study led to ultimate failure in clinical trials [3].

Amniotic fluids contribute to cushioning, hydration, and, most notably, providing immunity to growing embryos. We separated embryonic stem cells from chick early amniotic fluids (ceAF) from growing eggs at day 6–8, which is the optimum exponential growth stage for embryos. Several studies indicated the potent role of stem cell-based therapies as an effective source against cancer progression [4,5,6]. These studies revealed the use of neural stem cells isolated from the differentiation stage of embryonic tissues, human amniotic fluid mesenchymal cells, and even cancer stem cells as an effective remedy against several types of cancer.

In this study, we used different types of tumor cell lines, MCF7, BCaP37, and RKO, to investigate whether ceAF could mediate any anti-inflammatory response against the progression of the tumor. MCF7 and BCaP37 cell lines are breast cancer-derived cells, whereas RKO is a colon-mediated tumor cell line. Breast cancer accounts for 25% of all cancer cases worldwide; even in 2012, breast cancer was reported to cause 15% of all cancer-associated mortalities among women [7]. Colorectal carcinoma is one of the most common malignant tumors [8] in which metastasis and invasion are crucial properties of malignant colon cancer, leading to high chances of recurrence post-surgery and therapeutic approaches [9]. We studied the progression of these cancer-related cell lines. We used precise dosage-specific titers of ceAF to investigate its role in different signaling pathways, including mitochondrial transmembrane potential ΔΨm and poly (ADP-Ribose) polymerase signaling pathways, all of them recognized as the key mediators and signaling markers in tumor progression [10,11,12].

ceAF exhibited a potential anti-inflammatory role against different tumor cell lines compared with a regular liver cell line (HL7702). Specific critical components, including polo-like kinase 1 (PLK1), observed inhibition of mitosis progression kinase cdc25c in Western blot analysis; moreover, gene expression of specific proteins including downregulation of Bcl-xl, cyclin-D, cyclin-e, and COX-2 and upregulation of c-myc, BAX, and caspase-3 was reported, which indicates that ceAF is performing response mediated therapy against tumor cells. The in vivo data are also consistent with in vitro studies implying the optimum concentration of ceAF to be 5% *v*/*v* among all cell lines. All of them indicate the promising anti-inflammatory properties and therapeutic potentials of ceAF.

## 2. Materials and Methods

### 2.1. Chick Early Amniotic Fluid (ceAF) Preparation

ceAF was collected from fertilized eggs (6–8 days post fertilization, incubated at 38 ± 1 °C and 50% humidity). Briefly, amniotic fluid was collected from amniotic sac from the growing chicken embryo by 1 mL U-100 syringe and kept in 50 mL falcon tube on ice while collecting. Once the tube was full, the supernatants were centrifuged at 2500× *g* for 20 min and filtered over a 0.22 µm sterilization device (Millipore China). The aliquots were stored at 80 °C after quick-freezing in liquid nitrogen.

### 2.2. Cytotoxic Assay Using Cell Counting Kit (CCK8)

Cells (BCaP37, MCF7, RKO and HL7702) were dispensed 100 μL of cell suspension (5000 cells per well) in a 96-well plate. The plate was pre-incubated for 24 h in a humidified incubator (e.g., at 37 °C, 5% CO_2_). Different fractions of ceAF (20%, 10%, and 5% *v*/*v*) were added into the culture media in plate. The plate was incubated for the following intervals: 12, 24, 48, 72, 96, and 120 h. The CCK-8 (MedChemExpress^®^, Monmouth Junction, NJ, USA) solution was added, 10 μL, to each well of the plate. The plate was further incubated for three hours. The absorbance at 450 nm was measured using a microplate reader.

### 2.3. In Vitro Trans-Well Migration Assay

Cells were collected/suspended at a density of 3 × 10^4^ cells in 200 µL serum-free medium after treatment with or without different fractions of ceAF for 24 h and seeded in the upper chamber. Media with indicated doses of ceAF (600 µL in total) was suspended in the lower chamber. After 24 h, chambers were separated, and the cells on the upper surface of the membrane were wiped off with cotton buds. Cells that invaded the microporous membrane (8 µm diameter) were rinsed with PBS thrice, fixed with 4% paraformaldehyde for 30 min, and stained with 0.1% crystal violet. Number of cells that trans-well-migrated was observed/counted/recorded by a microscope (Olympus BX51).

### 2.4. Mitochondrial Transmembrane Potential Using Rhodamine 123

Following the protocol [13], approximately 1 × 10^5^ cells were seeded per well of a 12-well plate. After overnight incubation and post-12-h starvation, cells were treated with different fractions of ceAF for 24 h. For measuring mitochondrial potential and active proton gradients, cells were treated with rhodamine-123 dye (1 μg/mL) in the dark for at least 2 h. After fluorescent staining, cells were mounted on slides and observed under a confocal microscope (Olympus high-resolution confocal microscope, OSR).

### 2.5. Cell Cycle Analysis Using Aphidicolin

MCF7, BCaP37, RKO, and HL7702 cells were seeded in a 6-well plate with a seeding density of 1 × 10^6^. After starvation and treatment with different fractions of ceAF, cells were synchronized at the G1/S point using aphidicolin blocking [14]. Briefly exponentially growing cells were treated with aphidicolin (4 μg/mL) for 12 h and, later, washed thrice with PBS. Cells were treated with different fractions (*v*/*v*) of ceAF. Cells were detached using trypsin, and sediments were resuspended in a 1 mL hypotonic solution comprising propidium iodide (50 μg/mL) and RNAase solution. Cell cycle distribution was immediately analyzed by flow cytometry (DX-Flex, Beckman Coulter, Fullerton, CA, USA). Cells with less DNA content than G1 cells appeared as dead and apoptotic cells in cell cycle distribution. These cells were further analyzed by FlowJo (Becton, Dickinson, and Company, Franklin Lakes, NJ, USA; 2019) software.

### 2.6. Annexin-V Staining and Blocking for Apoptosis Detection

MCF7, BCaP37, RKO, and HL7702 cells were seeded in a 6-well plate with a seeding density of 1 × 10^6^. After starvation and treatment with different fractions of ceAF, using the protocol [15], cells were washed with cold PBS and resuspended in 1X binding buffer. Cells were later transferred to 100 μL of the solution (1 × 10^5^ cells) to a 5 mL culture tube, and 5–15 μg of recombinant Annexin V and propidium iodide (PI) was added. The cells were further washed with PBS twice before detecting FACS. FITC-positive and PI-negative cells were recognized as apoptotic cells.

### 2.7. Animals Testing and Caring

Pathogen-free mice BALB/c (5-week-old females, weighing between 20–25 g) were bought from SIPPR-BK Lab Animals Co. Ltd. Shanghai, China [Certificate # SCK (hu) 2013–0016]. The mice were divided and grouped randomly (5/cage) and kept in Zhejiang University Animal Center (Permit #: ZJU20170013). The cages were nurtured with a 12-hr day/light cycle, supplemented with rodent chow and water. All procedures were approved by institutional animal care facilities.

### 2.8. Xenograft Experiment

Female BALB/c nude mice were used for ceAF-treated modeling. Five-weeks-old mice were subcutaneously injected with ceAF-treated (20, 10, and 5% ceAF *v*/*v*) BCaP37, RKO, and MCF7 cell line 1.5 × 10^6^ cells on right flank. Tumor size was measured every third day after 10 days post-injection. Tumor length was calculated as (width^2^ × length × 0.5). Once the tumor was formed, the ceAF treatment was given systematically (intravenous) every third day. ceAF was dissolved as a buffered (pH 3.5) solution at the dosage of 40 mg/kg or vehicle. Treatment response was accessed according to the RECIST criteria [16]. Mice were sacrificed after 20 days post-tumor formation. Upon autopsy, xenograft tumors were stored in a 4% PFA solution until further use.

### 2.9. Histological Procedures

Excised tumor samples were fixed in 10% formalin for minimally five days and processed. Briefly embedded in paraffin, specimens were sectioned in 3 μm thickness, mounted on glass slides, and, after deparaffinized, stained with hematoxylin-eosin, Masson’s trichrome, and Ki67. The density of inflammatory cells and blood vessels in the dermis was analyzed using the M-42 system. Random fields of the tissue were observed and counted for particular sections with images taken by an optic microscope (Olympus, BX41, Tokyo, Japan).

### 2.10. RNA Extraction from Cells and cDNA Preparation

For isolating RNA from ceAF-treated cells, the cells were trypsinized and transferred in RNAse-free tubes following the protocol [17]. cDNA was prepared from isolated total RNA using a cDNA preparation kit. RNase-free double distilled water, 4X gDNA wiper mix, and 1 µg total RNA were consecutively pipetted into a tube and placed at 42 °C for 2 min. Later, 5X Hi Script Mix II was added, and the samples were placed at 50 °C for 15 min and 85 °C for 5 s before qPCR. cDNA was stored at −20 °C for future use.

### 2.11. Western Blotting

For immunoblotting, the cells were cultured and treated with different fractions of ceAF; later, the media was aspirated. Cells were washed with PBS twice and lysed with HEPES buffer (115 mM NaCl, 1.2 mM CaCl_2_, 1.2 mM MgCl_2_, 2.4 mM K_2_HPO_4_, 20 mM Hepes-KOH, pH 7.0, 1% NP40) added protease and phosphatase inhibitors. Samples were normalized for SDS-PAGE loading. Protein expression was also normalized with an internal control (β-actin). Signals were detected using primary antibodies followed by HRP-conjugated secondary antibodies.

### 2.12. Statistical Analysis

The data were represented as mean ± SEM, and statistical analysis was performed using Prism GraphPad (San Diego, CA, USA). One-way ANOVA and two-way ANOVA were used to determine significance. * *p* < 0.05, ** *p* < 0.01, and *** *p* < 0.001 were taken as statistically significant. All in vitro experiments were independently repeated in triplicates.

## 3. Results

### 3.1. ceAF Inhibited Cancer Cells Proliferation

**Figure 1:**
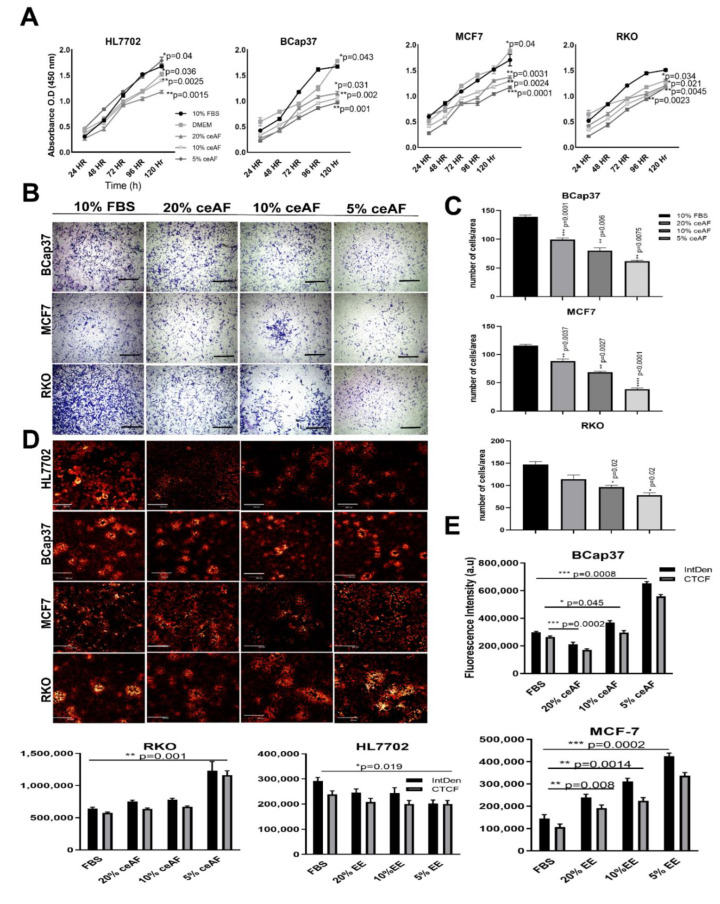
ceAF significantly downregulates tumor cells’ proliferation and growth via disrupting transmembrane potential. (**A**) Cytotoxicity assay of tumor cell lines BCaP37, RKO, and MCF7 induced by different fractions of ceAF on different time frames using cck8. The cell growth curve is shown on (**A**), whereas HL7702 is used as a normal cell line for reference for comparison. (**B**) In vitro trans-well migration assay was performed to validate the migration of cells. Scalebar represents 400 μm. The number of cells migrated/area is shown in (**C**) panel. Confocal fluorescence images of tumor cell lines with different fractions of ceAF to determine transmembrane potential using rhodamine 123 dye (**D**). (Scale bar at 50μm.) (Excitation, 559 nm; emission, 575–675 nm.) Relative integrated density and absorbance of fluorescent dye in the cells are shown on the right side (**E**). Significance was measured using a one-way ANOVA where Dunnett’s multiple comparisons test was used to compare control (10% FBS) with other groups. * *p* < 0.05, ** *p* < 0.01, and *** *p* < 0.001.

Given that ceAF comprises embryonic stem cells and stem cell-based therapies are used as effective anti-tumor strategic sources [18,19,20], we believed that ceAF could somehow mimic stem cells’ behavior in exhibiting anti-tumor properties. For this, we tested different doses of ceAF against the growth parameters of tumor cells and used a regular cell line as a reference to differentiate the growth parameters; ceAF, showed a time- and dose-dependent inhibitory effect on the growth of diverse tested tumor cell lines including breast and colon cancer cell lines (Figure 1A; the CCK8 cell viability assays to reveal the potential of cytotoxic activity of ceAF against tumor progression). We also tested whether ceAF treatment inhibits cells from migrating through the chambers as shown in Figure 1B. We found that a 5% fraction of ceAF inhibits tumor cell proliferation and migration of cells, consistent with the previous finding. This suggests anti-inflammatory properties associated with ceAF that, in theory, contain factors secreted by chicken embryonic stem cells. When tumor cells were dosed with different concentrations of ceAF, their potential to grow and proliferate was inhibited in a dosage-dependent manner (*p* < 0.05) prominently in the 5% ceAF group in all cell lines.

### 3.2. ceAF Treatment Reduces Cancer Cells Colonies and Disrupts Mitochondrial Transmembrane Potential

To assess whether ceAF exhibits any morphological differences in the colonies of tumor cells, we tested this by treating cells with different concentrations of ceAF and stained the cells with rhodamine 123 dye [21] which not only exhibits colony dissociating parameters, but also differentiates quiescent cells due to mitochondrial transmembrane potential [22]. The control groups have shown colony-forming abilities, whereas groups treated with ceAF showed a dose-dependent reduction in the colony-forming ability of cells. Colony deformation can be seen in RKO, B-CaP37, and MCF7 cell lines (Figure 1C, quantification of fluorescence intensity can be seen on the right side of the panel) compared to the control cell line HL7702. Treatment also effectively induced ultrastructural alterations in tumor cell lines. Relative integrated fluorescent densities of different cell lines can be seen as well.

### 3.3. ceAF Induces Cell Cycle Arrest and Apoptosis in Cancer Cells

**Figure 2:**
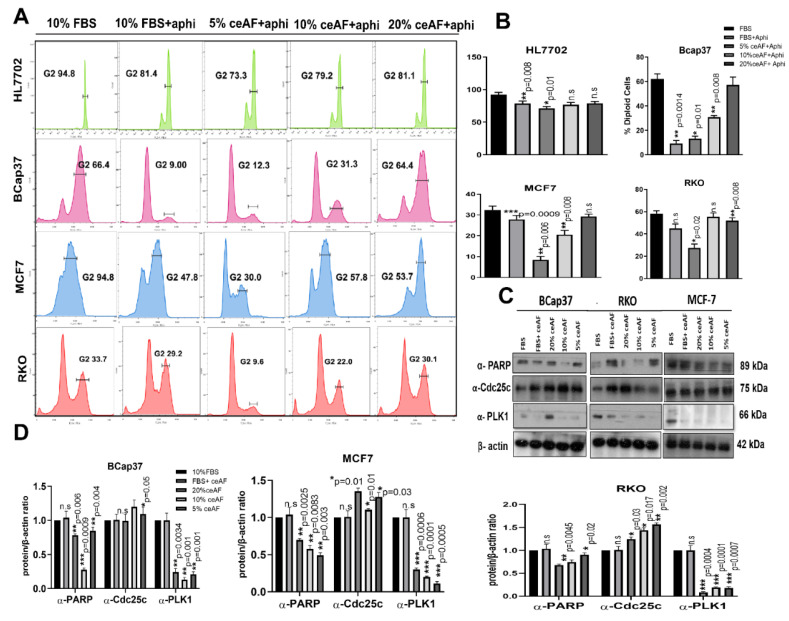
ceAF works agonistically with aphidicolin to arrest tumor cell metastasis. (**A**) Role of ceAF in suppressing growing tumor cells transition in cell cycle via arresting at G1 phase using aphidicolin. Cells were treated with different fractions, 20, 10, and 5% (*v*/*v*) of ceAF, and later treated with a reversible inhibitor of DNA aphidicolin. Flow cytometry measured DNA content by tracing cells’ fluorescence intensity. The percentage of diploid cells is shown on the right side (**B**), whereas the HL7702 cell line was used as usual. (**C**) Immunoblots showing significant upregulation of proteins regulating cell cycle and apoptosis (Figures S1–S3). In contrast, β-actin is used as an internal control. Quantification of protein bands was performed by densitometry, and the relative gene/actin ratio is shown in the bottom panel (**D**). The results shown here are representative of at least three independent experiments. The semi-quantitative analysis of blots is shown were Student’s *t*-test obtained mean ± SD * *p* < 0.05 and ** *p* < 0.01 and *** *p* < 0.001.

The effect of ceAF on cell cycle distribution was analyzed to understand the anti-inflammatory properties mechanism. Exposure of tumor cell lines to growth-suppressive doses of ceAF (10% and 5% *v*/*v*) resulted in blockage of significant transitioning of cells from the G1 to G2 phase, which is arrested in the negative control where cells were treated with aphidicolin only. This effective blocking of cells in the G2/M phase suggests the declined number of diploid cells, which can be seen in (Figure 2A). A distinct sub-G1 peak in the groups treated with ceAF offers the dead and apoptotic cells present because of aphidicolin treatment. The results indicate that ceAF could work under aphidicolin to block the proliferation of tumor cells in a dosage-dependent manner in vitro.

### 3.4. Regulation of Apoptosis and Cell Cycle-Related Protein Expression

Since ceAF was observed to cause cell cycle arrest and apoptosis, we further analyzed G2/M cell cycle regulators, including Cdc25c and polo-like kinase1. The results revealed that ceAF increased Cdc25c and inhibited polo-like kinase 1 expression. Since apoptosis is also associated with DNA damage, we observed any DNA damage control proteins in the tumor microenvironment. We analyzed the PARP protein linked with DNA repair, cell proliferation, and cell death. PARP reactions are needed for cells to progress through the G2/M phases of the cell cycle and prevent damaged cells from having DNA strand breaks from entering mitosis. Moreover, indicated sub-G1 peaks present during FACS analysis (Figure 2C,D).

### 3.5. ceAF Induces Apoptosis

**Figure 3:**
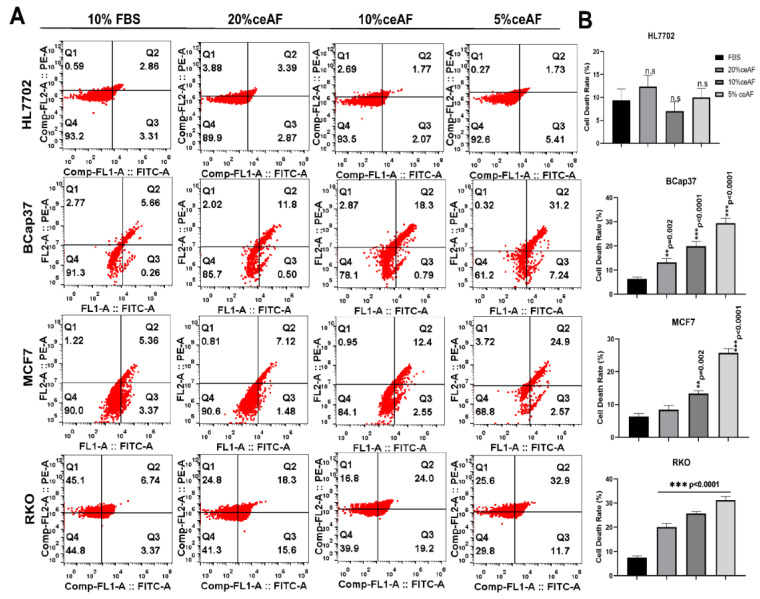
ceAF induces apoptosis of tumor cells via MAPK-ERK signaling as determined by annexin V-FITC signaling. (**A**) Tumor cells were treated with ceAF and, after synchronization, subjected to annexin v staining. Flow cytometry determined the percentage of apoptotic cells; relative quantification is shown in the right panel (**B**). Significance was measured using a one-way ANOVA where Dunnett’s multiple comparisons test was used to compare control (10% FBS) with other groups., ** *p* < 0.01, and *** *p* < 0.001.

The process of controlled cell death is known as apoptosis; it has a central role in maintaining cellular homeostasis and several other diseases, including neurodegenerative, cancer, cardiovascular, and auto-immune diseases [23]. After monitoring the cytotoxic and anti-inflammatory effects of ceAF, we further confirmed the mode of cell death by measuring annexin-V-conjugated mediated flow cytometry for apoptosis. The FACS results revealed a significant and distinct apoptotic population of treated cells compared to control groups in Figure 3. As shown in Figure 3A, quantification of the number of apoptotic cells is given on the right side of the panel; Figure 3B. Overall, our results confirmed the induction of apoptosis in tumor cells in a dose-dependent manner, confirming the possible therapeutic potentials of ceAF for recruiting tumor cells to undergo apoptosis.

### 3.6. Gene Expression Analysis

**Figure 4:**
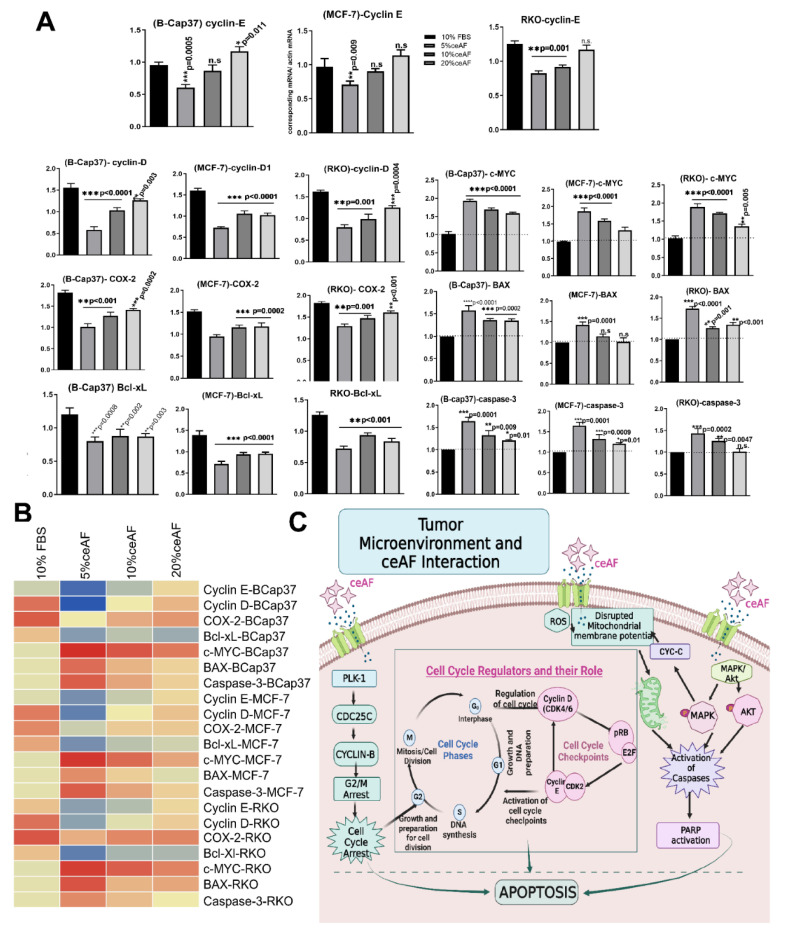
ceAF downregulates the pro-apoptotic genes in a dose-dependent manner. Quantitative real-time PCR analysis of tumor cells treated with different fractions of ceAF (5, 10, and 20% *v*/*v*) is shown. ceAF down-regulates cyclin D and E most effectively in the 5% ceAF group, which shows arresting a maximum number of cells at the G1 phase in the results above (**A**). A comprehensive summary of gene expression is shown in the heatmap (**B**) in the left bottom panel (**B**). All experiments were performed three times independently, whereas data represents as mean ± SD * *p* < 0.05 ** *p* < 0.001 and *** *p* < 0.0001. (**C**) ceAF signals tumor cells to undergo apoptosis via cell cycle arrest and MAPK-Akt signaling thereby restricting the growth and proliferation of cells. The declined expression of PLK-1 results in significant expression of CDC25c, which signals cyclin B (cell cycle checkpoint) to halt the G2/M transition of the cell cycle. This also checks and arrests the cells at different phases of the cell cycle-regulated by other checkpoints, including cyclin D and cyclin E. The further possible activation that ceAF may regulate is the activation (phosphorylation) of MAPK-Akt signaling pathways, which activates a series of caspases to undergo apoptosis. Moreover, ceAF regulates the expression of reactive oxygen species (ROS) in the tumor microenvironment, which can disturb the mitochondrial membrane potential (ΔΨm), thereby rupturing the tumor cells’ morphology.

Relative expression of apoptosis regulatory genes was measured by quantitative real-time PCR. Since ceAF has induced apoptosis in the tumor microenvironment, we examined the effect of ceAF on the expression levels of some critical NFκB targeted genes, including cyclin E, Bcl-xL, cyclin D1, and COX-2 on cancer cell lines (the expression levels of these genes were significantly down-regulated). During cell growth and survival, the critical role is played by cyclins D1 and E [24,25,26]. As a result, down-regulation of their expression would certainly shift towards cell cycle arrest in the groups treated with ceAF (Figure 4A). COX-2 inhibition is also co-related to inducing apoptosis [26]. This result was further supported by regulating anti-apoptotic Bcl-xL gene expression, a negative looped regulator of the effector caspase-3 [27]. It is also known as a downstream target of NFκB. Similarly, the up-regulation of caspase-3, an enhancer of programmed cell death, further verifies apoptosis in treated cells. A further important up-regulated gene was c-myc, a known regulator of p53 [28]. C-myc induced p53 which possibly up-regulated the other pro-apoptotic gene BAX gene. The findings above imply the critical role of ceAF, which is essential in inducing apoptosis in tumor cell lines. A comprehensive summary of the relative gene expression of all cell lines is shown in the heatmap (Figure 4B). A possible detailed description of how ceAF contributes to deterring tumorigenesis via cell cycle arrest and disrupting mitochondrial membrane potential is shown in Figure 4C.

### 3.7. ceAF Treatment Significantly Inhibits the Malignant Tumor Phenotype of Cancer In Vivo

**Figure 5:**
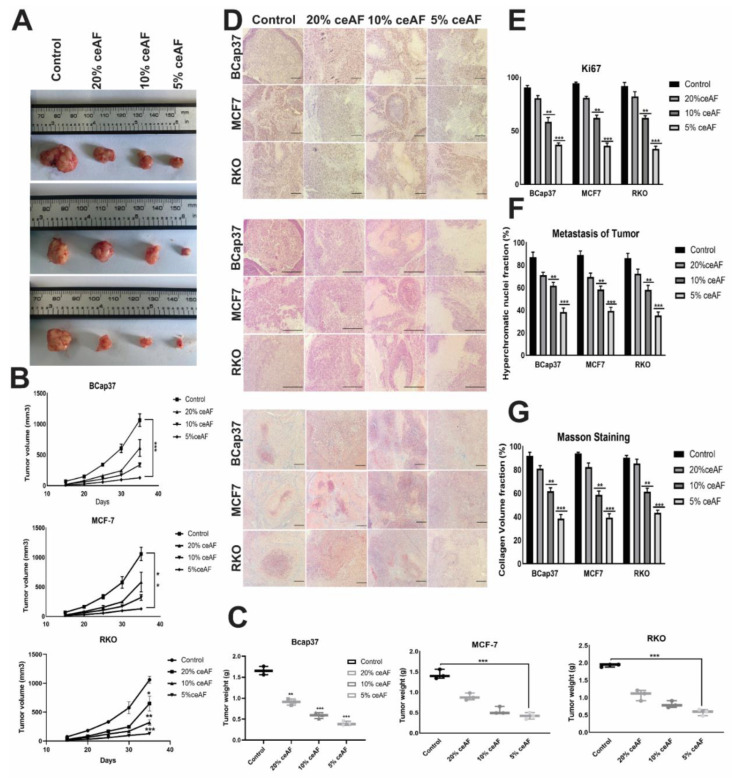
ceAF significantly inhibits tumor metastasis in the xenografts model (in vivo). The effect of ceAF in tumor growth inhibition in nude mice. Tumor size and tumor weight of mice treated with different fractions of ceAF (20, 10, and 5 *v*/*v*) compared to the control group (cells inoculated without giving any treatment) (**A**). Body weight and tumor volume (mean ± SD) of nude mice are shown (**B**,**C**). Representative staining images of H&E, Ki67, and Masson Trichrome in tumor xenografts (**D**). Significantly large necrotic areas in the 5% ceAF treated group can be seen as indicated via H&E staining. Decreased expression of Ki67 in the tumors treated with 5% ceAF mice can also be seen compared with control (**E**). In vivo inhibition of angiogenesis and increased necrosis with ceAF treatment was observed and validated via Masson’s trichrome staining (**F**). Density of collagen fibers per unit area (percentage) among different groups can be seen in (**G**). A relative quantification of nuclei fraction, number of positive Ki67 stained cells, and collagen volume fraction are shown in the extreme right panel, whereas statistical significance was evaluated with a two-way ANOVA with Bonferroni post-test. (N = 3/group) where, * *p* < 0.05, ** *p*, 0.001 and *** *p* < 0.001. scalebar represents 100 μm.

We also ascertained the influence of ceAF treatment and its different fractions (*v*/*v*) on nude mice (BALB-c). The tumor volume of the groups treated with 5% ceAF on other cell lines was smaller than the control group (cells were generally seeded in 10% serum and then injected subcutaneously). Similarly, the mice group treated with 10% ceAF had relatively small tumor volume compared with the control group indicating promising anti-inflammatory properties of chick early amniotic fluid. As shown in Figure 5A. The tumor weight and volume quantification can be seen in the bottom panels (Figure 5B,C).

### 3.8. Characterization of Tumor Models

#### 3.8.1. H&E Staining

Tumors treated with different fractions of ceAF exhibited significantly different results compared with the control group. as H&E staining results indicate, there were significantly large necrotic areas in the 5% ceAF-treated group. Interestingly, the groups treated with ceAF (particularly 5% and 10% ceAF) exhibited focal sarcomatous phenotype with spindle cells and poorly differentiated cells compared to control groups in all cell lines. The staining images of BCaP 37 grouped mice can be seen in Figure 5D.

#### 3.8.2. Masson’s Trichrome Staining

The vascularity of tumor-implanted cells in mice was histologically evaluated using Masson’s trichrome staining. Tumors from ceAF-treated cells showed relatively reduced blood vessels compared to tumors excised from vehicle-treated mice. Moreover, in vivo inhibition of angiogenesis and increased necrosis with ceAF treatment was observed and validated via Masson’s trichrome staining, which can be seen in Figure 5D–G.

#### 3.8.3. Ki67 Staining

Ki67 is known to be a key marker of tumor proliferation. We fixed our xenograft tumors with formalin and embedded them in paraffin, followed by immunohistochemistry to visualize the expression of this marker. Representative images of Ki67 are shown in Figure 5, indicating the decreased expression of Ki67 in the tumors treated with 5% ceAF mice compared with the control.

## 4. Discussion

In the present study, several observations have been made concerning the therapeutic efficacies of ceAF against tumor progression. Our previous work, [29], determined the ceAF’s therapeutic potential in healing cutaneous wounds. We found the role of transient senescence intimately merged with senescence associated secretory phenotypes (SASP), which functions collaboratively with ceAF for facilitating wound healing. Thus, exploring the therapeutic roles of ceAF, we tested the same fractions of ceAF against proliferating solid tumor cell lines to determine whether it plays any role against tumorigenesis. We selected three cell lines: BCaP37, MCF7, and RKO. Moreover, a regular cell line (HL7702) was used as a reference for comparing the tumor cell lines against the control. We performed different biochemical assays, which are considered markers for monitoring the tumor progression/inhibition, including cell cycle arrest using a G-phase inhibitor drug, cytotoxic assays, mitochondrial transmembrane potential, and quantitative real-time PCR, in-vivo experiments and protein expression profiles..

The cytotoxicity assay using CCK8 revealed the cumulative inhibition of cell proliferation in all cell lines, but in a dosage-dependent manner, indicating the efficacy of ceAF while emphasizing the fact that critical concentration is inevitable for effective treatment against cancer, shown in Figure 1A, which was further confirmed via in vitro trans-well migration assay. ceAF induced apoptosis in different tumor cells, which was validated by annexin-V staining (Figure 3A) and rhodamine 123 staining (DNA fragmentation and colony deformation) (Figure 1D), which was further verified by the caspase 3 gene expression profile, which was consistent in all cell lines (quantitative real-time PCR; Figure 4). The critical step in the mitochondria-mediated apoptotic pathway is disrupting the mitochondrial membrane, eventually causing loss of ΔΨm. In our study, ceAF decreased ΔΨm and disrupted the sequential colonies formed by tumor cells, possibly triggering the apoptosis signaling pathways (Figure 1D). The inhibition of the proliferating ability of tumor cells and the induction of apoptosis is associated with the activation of other intracellular signaling pathways as indicated in cell cycle arrest in the S, G1, or G2/M phase [29,30]. Our results suggest that cyclin-D and cyclin-E could play a potential role in ceAF-mediated G2/M phase arrest (quantitative real-time PCR; Figure 4) as verified by conjugated cell cycle arrest using aphidicolin, resulting in the restriction of possible apoptotic cell death and cell growth (Figure 2A).

Amniotic fluids, stem cells, and mesenchymal cells have promising roles as stem cell-based therapies for some life-threatening human diseases [31]. Furthermore, these fluids have low immunogenicity, anti-inflammation, and non-inflammatory features. Our previous studies also demonstrate the promising therapeutic potential of ceAF against cutaneous wound healing and myocardial infarctions [32,33]. Several in vivo experiments show that their tumor tropism has opened new horizons for cancer cell therapies [34]. To support our findings on the positive role of ceAF against tumor recurrence and inhibiting the proliferation of tumor cells, we inoculated the ceAF-treated cells in nude mice and treated the mice with ceAF via subcutaneous injection once the tumor manifested. To our surprise, the 5% ceAF group among all cell lines inhibited tumor growth via suppressing the proliferation and induced apoptosis, as shown in Figure 5A; tumor volume and weight are also shown.

The downregulation of specific oncogenic genes, including cyclin-E, Bcl-xL, cyclin-D1, and COX-2 (Figure 4), indicated anticancer properties of ceAF with concomitant up-regulation of c-myc, caspase-3, and BAX. ceAF caused inhibition of proliferation, G2/M (Figure 2) phase arrest, and induction of apoptosis which led to activation of caspase-3 and BAX. Apoptosis signaling systems are suitable targets for developing new anticancer agents [35]. Down-regulation of cyclin-E, cyclin-D, and Bcl-xl and upregulation of the expression of pro-apoptotic proteins Bax and C-myc could play a critical role in ceAF potential to stimulate apoptosis in MCF7, BCaP37, and RKO cell lines, however, the downregulation of expression of COX-2 is probably due to anti-metastatic effects of ceAF, which might require further research. P53-mediated cell cycle arrest and apoptosis also effectively remedy tumor progression. It can result in G1-mediated cell cycle arrest by targeting down streaming of the p21 gene, which is mentioned in early research [36]. In our case, ceAF appeared to induce G2/M arrest in cells to post 24 h of treatment. Several signaling pathways control and facilitate these cell cycle events [37]. Among them, many regulatory enzymes have a remarkable role, including polo-like kinase-1 (PLK-1), which is identified as a critical player in G2/M transition and mitotic progression in both tumor and normal cells. PLK-1 mediates several mitotic processes, including activation of Cdc25c, bipolar spindle formation, centrosome maturation, actin ring formation, and activation of anaphase-promoting complex [38,39]. As indicated by Western blots, the decreased expression of PLK-1 resulted in the significant accumulation of Cdc25c (Figure 2C). Our data confirmed the hypothesis of down-regulation of PLK-1-induced mitotic arrest following apoptosis via the Cdc25c/cdc2/cyclin B1 feedback loop [40]. ceAF induced apoptosis not only through MAPK-Akt signaling and cyclin-E mediated cell cycle arrest, but also via destabilization of lysosomal membranes, which further leads to mitochondrial membrane depolarization and activation of caspases [41]. Moreover, the internal signal pathway of apoptosis has been considered a sequential event of mitochondria changes, majorly the decline of ΔΨm and activation of the caspase cascade [42,43]. Amniotic fluids, stem cells, and mesenchymal cells have promising roles as stem cell-based therapies for some life-threatening human diseases [44].

Furthermore, these fluids have low immunogenicity, anti-inflammation, and non-inflammatory features. Several in vivo experiments show that their tumor tropism has opened new horizons for cancer cell therapies [34]. To support our findings on the positive role of ceAF against tumor recurrence and inhibiting the proliferation of tumor cells, we inoculated the ceAF-treated cells in nude mice and treated the mice with ceAF via subcutaneous injection once the tumor manifested. To our surprise, the 5% ceAF group among all cell lines inhibited tumor growth via suppressing the proliferation and induced apoptosis, as shown in Figure 5B,C; tumor volume and weight are also shown. Furthermore, H&E staining, Masson’s trichrome staining, and Ki-67 staining of xenografts biopsies also show consistent results indicating promising potentials of ceAF against tumor suppression in a dosage-dependent manner.

## 5. Conclusions

In conclusion, our study implies the therapeutic efficacies of ceAF, which possess a balanced fraction of growth factors, stimulants, inhibitors, and cytokines that have the potential to be used as an effective therapeutic source against tumor progression. Hence, several apoptotic signaling pathways act simultaneously as a feedback mechanism in protecting the cellular environment against unwanted and uncontrolled proliferation of tumor cells. Further studies and research are required to disclose the exact composition of ceAF so that specific targeting-based strategies can be conducted to evaluate and screen apoptotic pathways and how we can use this therapeutic source at the next level of in vivo studies in preclinical trials.

## Data Availability

Not applicable.

## Supplementary Materials

The following supporting information can be downloaded at: https://www.mdpi.com/article/10.3390/biology11111577/s1, Figure S1: BCaP37 Western Blotting; Figure S2: MCF-7 Western Blotting; Figure S3: RKO Western Blotting.
