# Supplementary material for: Chick Early Amniotic Fluid (ceAF) Deters Tumorigenesis via Cell Cycle Arrest and Apoptosis
Source: Biology (Basel). 2022 Oct 27;11(11):1577. doi: 10.3390/biology11111577 (PMC9687777; doi:10.3390/biology11111577)
Supplement: Supplementary file 1 [file biology-11-01577-s001.zip › biology-1972698-supplementary.pdf]

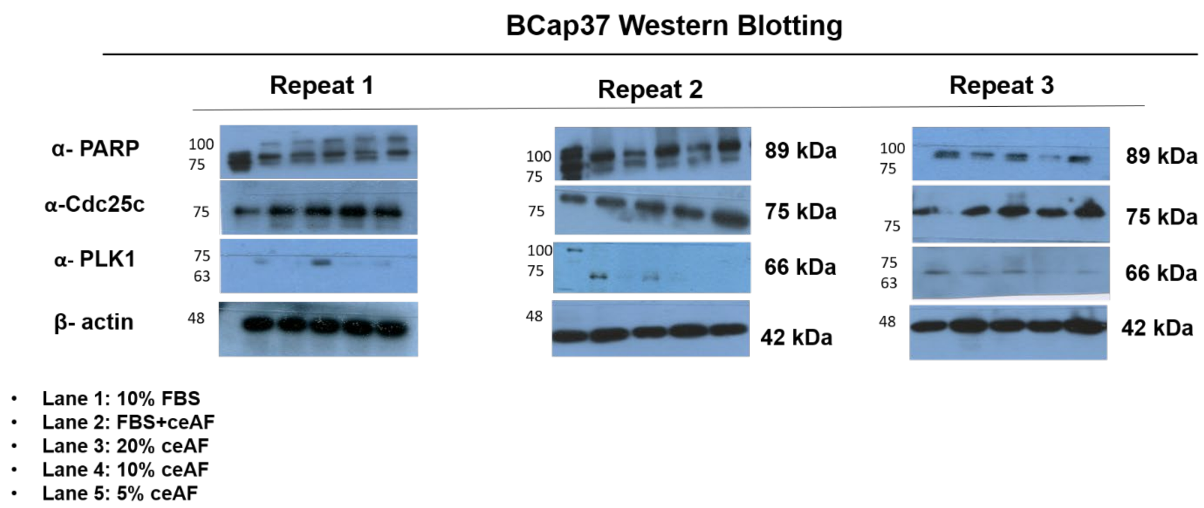

Figure S1: BCaP37Western Blotting.

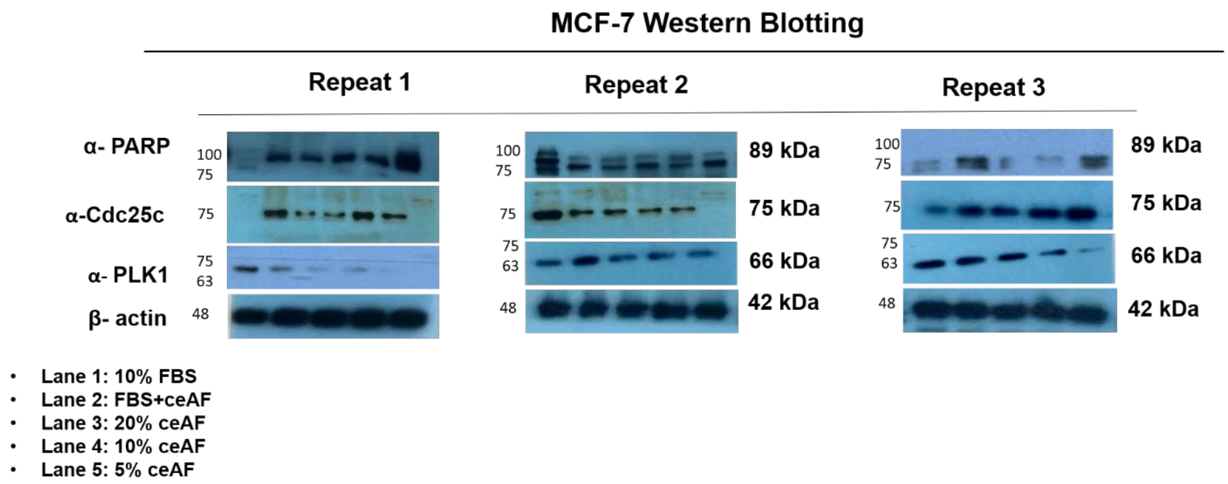

Figure S2: MCF-7Western Blotting.

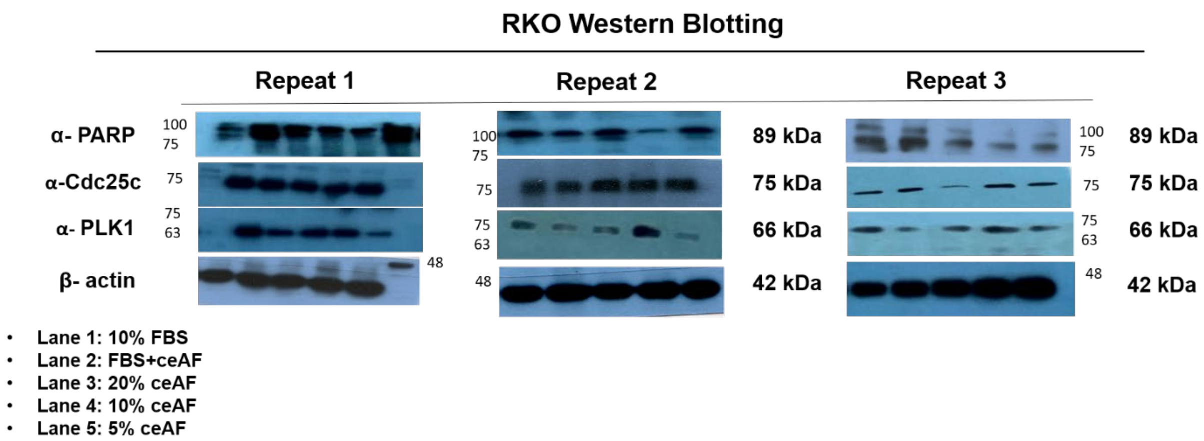

Figure S3: RKO Western Blotting.
